# Psychotherapeutic Change in Intensive Day Treatment for Personality Disorders: A Single Case Study of Quantitative and Qualitative Change in Agency and Communion

**DOI:** 10.1002/pmh.70007

**Published:** 2025-02-11

**Authors:** Silvia M. Pol, Elke Brok, Gerben J. Westerhof

**Affiliations:** ^1^ Scelta, Expertise Center for Personality Disorder GGNet Netwerk voor Geestelijke Gezondheidszorg in Oost‐Gelderland en Zutphen Netherlands; ^2^ Nijmegen, Karakter Netherlands; ^3^ Department of Psychology, Health, and Technology University of Twente Netherlands

**Keywords:** agency and communion, narrative identity, personality disorders, psychotherapeutic change, ROM, single case study

## Abstract

A better understanding of psychotherapeutic change is seen as essential for further development of treatment for personality disorders. The objective of this study is to describe the psychotherapeutic change process of a client with personality disorder to develop more insight in psychotherapeutic change processes. The change process was described quantitatively from ROM data and quantitatively and qualitatively from two narrative themes, agency and communion, described from the perspectives of client and treatment team. Reliable change analyses showed decrease in personality problems and increase in personality functioning and mental well‐being. Content analyses from the client perspective showed positive changes in meaning, actual behavior change, and connection with others. The treatment team noticed growth in self‐management ability and in connecting with own emotions and with others. These changes resulted in an increase in agency and communion. By mapping change processes through multiple sources and perspectives, the efficacy of psychotherapeutic treatment can be better understood.

## 1 Introduction

Personality disorders (PDs) cause significant suffering and chronic problems and are linked to suicide risk and high economic costs (APA 2013; Soeteman et al. 2008). The Alternative Model for Personality Disorders (AMPD) diagnose PD based on personality dysfunction and pathological traits (APA 2013). The first criterion, the level of personality functioning (LPF), is defined by disturbances in self (identity and self‐direction) and interpersonal (empathy and intimacy) functioning. Because of the struggle with the mental representation of self in relation to others, it is a major challenge for persons with PD to assign meaning to their own experiences, to direct their own lives, and to enter into relationships in a complex and constantly changing world.

Psychotherapy is the preferred treatment for PD (Simonsen et al. 2019). There is evidence for efficacy of psychotherapy (Binks et al. 2006; Budge et al. 2013; Cristea et al. 2017; Stoffers et al. 2013), yet a better understanding of psychotherapeutic change is considered essential for the further development of treatments for PD (Gunderson 2018; Kadzin 2007; Kealy and Ogrodniczuk 2018). Livesley, Dimaggio, and Clarkin (2016) describe two mechanisms of change specifically relevant to working with PD: a relational approach with an emphasis on repairing contact ruptures and fostering mentalizing ability, that is, understanding the mental state of oneself and others. Dimaggio et al. (2018) add the enrichment of self‐narratives as a third mechanism of change: the ability to retrieve rich and nuanced autobiographical memories that correspond to subjective experiences. The quality of self‐narratives is important in developing a coherent and stable narrative identity, which is associated with mental health and psychological well‐being (Adler 2012). Narrative identity is seen as central to the development of identity (Sharp and Wall 2021) and refers to the internalized and evolving story of the self, which an individual constructs through self‐reflection and meaning‐making of their own life (McAdams 2001). In a systematic review, Lind, Adler, and Clark (2020) focused on how people with PD and personality pathology construct their narrative identity. Some of the most characteristic aspects of their narrative identity were found to be low agency and low fulfillment of communion, referring to the extent to which communal needs are met (Adler 2012).

When it comes to better understanding psychotherapeutic treatment, the dimensions self and interpersonal functioning provide a good basis for research, corresponding to the two central themes in narrative identity: agency and communion (Adler et al. 2012; Lind, Adler, and Clark 2020; McAdams et al. 1996). Agency refers to the degree to which individuals are able to affect change in their own lives or influence others in their environment, often through demonstrations of self‐mastery, empowerment, achievement, or status. Communion refers to the degree to which individuals demonstrate or experience interpersonal connection through love, friendship, or dialog (McAdams and McLean 2013). The positive qualities of both dimensions are strongly associated with well‐being (Guisinger and Blatt 1994; Ryff and Singer 1998; McAdams 2001) and are considered essential in all major personality theories (Guisinger and Blatt 1994). However, second wave positive psychology has drawn the attention to the phenomenon of underuse and overuse of positive qualities (Lomas and Ivtzan 2016) and the resulting negative impact on the person self and others (Niemiec 2019). Therefore, promoting agency and communion is seen as the primary goal of treatment (Williams and Levitt 2007), but in doing so, guidance is needed to discover the optimal use of these qualities (Niemiec 2019).

Many studies examined agency and communion as narrative themes in PD. For example, Adler et al. (2012) found that presence of agency and fulfillment of communion (but not communion per se) in life story interviews correlated negatively with borderline personality disorder (BPD) criteria. Lind et al. (2019) described an increase in agency, whereas other aspects of BPD patient's own and their parents' life stories, that is, communion and communion fulfillment, did not change after therapy. In another study, Lind et al. (2023) found an increased focus on agency (instead of communion) in narrated events in adolescents with BPD throughout MBT group therapy. In life stories of patients with PD after therapy, Pol et al. (2023) found an increase in agency, but not in communion as a whole because of a decrease in the number of relationships. However, there was a significant increase in perceived quality of relationships. Timberlake and Fesel (2023) showed that agency and coherence improved throughout therapy with a patient with avoidant PD whereas communion fulfillment declined. After 6 months, both agency and communion fulfillment had increased, whereas coherence remained unchanged.

The results of these studies suggest that improvement in perceived ability to pursue goals, develop skills and achieve performance (agency), and form and maintain social connections (communion) (e.g., Abele et al. 2008; Ybarra et al. 2008) are important factors contributing to recovery from PD and personality pathology. This perspective corresponds to a person‐centered approach that shifts the focus from symptoms and limitations to personal recovery, in which rebuilding and defining a positive sense of identity is one of the key processes (Leamy et al. 2011). To this means, clients are encouraged to explore and articulate their personal stories as a means of fostering self‐understanding and personal growth (Haselberger and Hutterer 2013). Consistent with a person‐centered approach is a treatment evaluation that values clients' stories and experiences and also allows the practitioner to evaluate the treatment relationship and process (Hafkenscheid 2021). In this regard, single‐case methodology offers the opportunity to delve deeper into the course of treatment from the perspective of client and practitioner and can therefore contribute to a better understanding of change processes.

In this contribution, we present a single case study of an intensive psychotherapeutic treatment process based on both quantitative and qualitative source material. In addition to outcomes on routine outcome monitoring (ROM), we study the perspective of a client and the treatment team based on existing narrative data. In a mixed methods approach, we use multiple sources to capture treatment efficacy in order to develop a better view of the change processes that occur during treatment. These include changes in (1) general level of complaints and personality functioning at the beginning of treatment to the follow‐up after 1 year and (2) changes in the narrative themes of agency and communion in (a) the life story at the beginning and after treatment, (b) the client's perspective, and (c) the treatment team's perspective.

## 2 Method

### 2.1 Setting

This study was conducted at a psychotherapeutic day clinic for PD at Scelta‐GGNet, the Netherlands, focusing on behavioral change to improve personal and social functioning. Clients participated in a 9‐month program, attending 3 days a week, which included group psychotherapy, art therapy, music therapy, psychomotor therapy, social reintegration, and relationship and family therapy. The program targeted clients with emotion regulation problems, including suicidality and self‐harm, and social and community functioning issues. Previous treatments had been insufficient. Contraindications included psychosis, substance dependence, intellectual disability, insufficient Dutch language proficiency, or antisocial PD as the main diagnosis.

### 2.2 Client

At intake, a young woman in her late twenties exhibited extreme self‐criticism, negative thinking, and obsessive‐compulsive behaviors related to eating and exercising. Her background included a troubled childhood with parents who had serious mental problems. She had dropped out of university and avoided social contact, unable to handle her emotions, feeling insecure, and believing others thought negatively of her. Despite this, she hid her anxiety behind a confident facade. Over 10 years, she had undergone various outpatient and inpatient treatments for anxiety, eating problems, and burnout.

Based on SCID‐I and SCID‐II interviews (van Groenestijn et al. 1999; Weertman, Arntz, and Kerkhofs 2000) at intake, the client met criteria for major depressive disorder (recurrent, moderate), panic disorder without agoraphobia, and obsessive‐compulsive PD. Clinical examination added borderline and avoidant traits due to severe self‐pathology and emotion dysregulation. The client was not using psychopharmaceuticals before or during treatment.

### 2.3 Procedure

#### 2.3.1 Selection Process

This case was selected based on the availability of a life story before and after treatment, three treatment evaluations, and complete ROM data. Willingness to complete a follow‐up measurement was also required. From a previous study (Pol et al. 2023), 34 life stories were available, with 16 from this day clinic treatment. Six cases had both narrative and complete ROM data. This case was chosen randomly, ensuring sufficient reflection in treatment evaluations for meaningful analysis. It represents severe, therapy‐resistant problems but is less representative in terms of ROM questionnaire completion.

#### 2.3.2 Informed Consent

The data collected prior to and during treatment were part of the regular practice in the intake procedure and treatment program. For a previous study (Pol et al. 2023), permission was sought from the client to use this preexisting data as well as her life story after treatment. For this study, her cooperation was sought to supplement this data with a posttreatment measurement.

#### 2.3.3 Process Measurements

A total of six ROM measurements were taken; the baseline measurement took place 1 week after start of treatment (T0), the second measurement at 6 weeks (T1), the third measurement at 18 weeks (T2), the fourth measurement at 30 weeks (T3), the fifth measurement at the end of treatment (T4), and the sixth measurement 12 months after the end of treatment (T5). No interim feedback was provided to the client or treatment team.

#### 2.3.4 Measuring Instruments

The Outcome Questionnaire 45 (OQ‐45; de Jong and Nugter 2004) assesses general psychological functioning, with high scores being indicative of lower performance. The Mental Health Continuum–Short Form (MHC‐SF; Lamers et al. 2011) is developed to measure positive mental health. The Severity Indices Personality Problems–Short Form (SIPP‐SF; Verheul et al. 2008) measures severity and changes in functional and dysfunctional personality functioning during treatment, with higher scores indicating a higher level of functioning.

#### 2.3.5 Narrative Data

The narrative data quotations were translated into English by the authors and double‐checked using DeepL Translator.

##### 2.3.5.1 Life Stories

The client wrote her life story in response to an open‐ended question before treatment: “Describe your life story, symptoms, previous treatment experiences, and expectations for intensive psychotherapeutic treatment.” After treatment, she was asked to write her life story again from her current perspective. These questions were designed to allow clients to freely express their experiences without a word limit (Pol et al. 2023).

##### 2.3.5.2 Psychotherapy Process, Client Perspective

The client's perspective was documented from treatment evaluations and analyzed for agency and communion themes (Abele and Wojciszke 2007). The client wrote evaluations after 6, 18, and 33 weeks, responding to the question: “Describe your progress in treatment in no more than one page, based on your treatment goals.”

##### 2.3.5.3 Psychotherapy Process, Treatment Team Perspective

The treatment team's perspective was documented through the intake report, two treatment‐team conclusions, and the posttreatment discharge letter, analyzed for agency and communion themes (Abele and Wojciszke 2007). The main practitioner wrote conclusions after 6 and 18 weeks, based on multidisciplinary team meetings reviewing the client's treatment goals. After 33 weeks, an evaluation meeting was held instead of a written conclusion.

### 2.4 Quantitative Analyses

#### 2.4.1 Measurement Tools

To examine pretreatment to posttreatment changes on self‐report lists, reliable change was calculated using Jacobson and Truax's (1991) equation:

RC=X2−X1⁄sqrt2*SE^2,

$$\mathrm{SE}={\mathrm{SD}}^{*}\text{sqrt}\left(1-r\right),$$

where (*X*1 − *X*2) is the difference between initial and follow‐up test scores and (*SE*) is the standard error of measurement, with (*SD*) as the standard deviation and (*r*) as the reliability coefficient. Internal consistency (Cronbach's alpha) was used as the reliability coefficient. The *SD* and *α* values were taken from similar studies (OQ‐45 and MHC‐SF; Franken et al. 2018, SIPP‐SF; Rossi, Debast, and van Alphen 2016). The MHC‐SF was the only questionnaire not administered before treatment as it was added to the ROM later.

#### 2.4.2 Narrative Data

Atlas.ti 8 software supported manifest content analysis (Hsieh and Shannon 2005) to systematically quantify data units. Two researchers (SP and EB) assigned meaning units, consisting of related words, phrases, or paragraphs (Graneheim and Lundman 2004). These units were hierarchically coded based on Pol et al. (2023) into themes of agency and communion, divided into seven subthemes: (1) presence of agency, (2) lack of agency, (3) excess of agency, (4) presence of maladaptive coping, (5) presence of communion, (6) lack of communion, and (7) excess of communion.

### 2.5 Qualitative Analysis of Narrative Data

A holistic content analysis (Lieblich, Tuval‐Mashiach, and Zilber 1998) was used to describe and interpret the narrative data. The steps included (1) reading the data units carefully and empathetically; (2) describing overall impressions and noting exceptions, contradictions, and disharmony; (3) following predetermined themes of agency and communion (Diehl, Owen, and Youngblade 2004; Abele et al. 2008); (4) highlighting and tracking these themes in the text; (5) writing conclusions; and (6) reaching consensus on theme emergence through discussion.

## 3 Results

### 3.1 Quantitative Analyses

#### 3.1.1 Measurement Instruments

There was a significant positive change in general functioning, positive mental health, and personality functioning at the end of treatment and at 1‐year follow‐up (see Table 1). Based on these data, it can be concluded that the client was successfully treated and that the psychotherapeutic treatment proved to be effective.

**TABLE 1:** ROM scores and reliable change (RC) during treatment and after 1‐year follow‐up.

	Score						*D*	
Vragenlijst	*T*0	*T*1	*T*2	*T*3	*T*4	*T*5	*T*0*–T*4	*T*0*–T*5
OQ‐45 (range 0–180)								
General psychological functioning	83	82	56*	43	58	52	−25*	−31*
	*T*0	*T*1	*T*2	*T*3	*T*4	*T*5	*T*1*–T*4	*T*1*–T*5
MHC‐SF (range 0–5)								
Positive mental health	—	2.71	3.36	3.71	3.71	3.93	1.00*	1.22*
Emotional well‐being	—	2.67	3.67	3.67	3.33	4.00	0.66	1.33*
Social well‐being	—	2.60	4.20*	4.00	4.00	4.20	1.40*	1.60*
Psychological well‐being	—	2.83	2.50	3.50	3.67	3.67	0.84	0.84
	*T*0	*T*1	*T*2	*T*3	*T*4	*T*5	*T*0*–T*4	*T*0*–T*5
SIPP‐SF (range 12–48)								
Self‐control	39	40*	37**	43*	45*	39*	6*	5*
Identity integration	32	32	33*	37*	33**	37*	1*	5*
Responsibility	40	44*	46*	40**	43*	43	3*	3*
Relational capacities	23	30*	29**	35*	33*	38*	10*	15*
Social concordance	45	47*	42**	46*	46	43**	1*	−2**

Abbreviations: *α*, Cronbach's alpha; D, difference in score; MHC‐SF, Mental Health Continuum–Short Form; OQ‐45, Outcome Questionnaire 45; SD, standard deviation; SIPP‐SF, Severity Indices of Personality Problems–Short Form; T4, end of day clinical treatment; T5, follow‐up measurement 1 year after end of day clinical treatment.

* Significant positive reliable change (RC > 1.56).

** Significant negative reliable change (RC > 1.56) both compared to the last measurement unless stated otherwise as in the last two columns.

#### 3.1.2 Narrative Data

Quantitative content analysis reveals the prevalence of themes in life stories, treatment evaluations, and team documents (see Table 2). The life stories before (1027) and after treatment (1150) were almost equal in length. In the life story after treatment, the client looks back on the past; lower agency and higher maladaptive coping here may indicate increased recognition of difficulties and understanding of her past functioning. Treatment evaluations show a gradual increase in agency and decrease in maladaptive coping, indicating positive therapeutic outcomes. Communion significantly increases during treatment. In the intake letter, treatment team conclusions and discharge letter, the team describe initially varying but eventually increasing agency and communion, and decreasing low agency.

**TABLE 2:** Themes and percentages of presence subthemes in life stories before and after treatment, treatment evaluations of the client during treatment, and intake report, treatment team conclusions, and discharge letter written by the team.

Theme	Autonomy				Communion		
Subtheme	AA	AG	AO	MC	CA	CG	CO
Datatype	%	%	%	%	%	%	%
Life Story 1	31.7	30.7	1.0	6.9	15.8	11.9	2.0
Life Story 2	23.7	29.0	0.9	16.1	18.6	12.7	0.0
Treatment Evaluation 1	50.0	22.7	0.0	15.9	11.4	0.0	0.0
Treatment Evaluation 2	66.0	20.8	0.0	7.5	5.7	0.0	0.0
Treatment Evaluation 3	38.2	6.6	0.0	10.5	43.4	1.3	0.0
Intake report	26.5	38.2	2.2	8.8	13.2	11	0.0
Team Conclusions 1	19.6	26.8	3.1	14.4	21.7	14.4	0.0
Team Conclusions 2	38.9	12.5	0.0	4.2	23.6	18.1	2.8
Discharge Letter	36.7	24.4	2.2	7.8	18.9	10	0.0

Abbreviations: AA, autonomy present; AG, autonomy, lack of; AO, autonomy, excess of; CA, communion present; CG, communion, lack of; CO, communion, excess of; MC, maladaptive coping.

### 3.2 Qualitative Analysis of Narrative Data

#### 3.2.1 Agency

##### 3.2.1.1 Life Story Before Treatment

In her early life, many events happen to the client over which she has no control. This made her anxious and insecure as a child. “When I found situations scary, I would run away. I ran away very often in my childhood.” From secondary school age onwards, the image emerges of a powerful girl and then a strong woman who knows how to stand up for herself and others. Social milestones seem to determine the storyline. She obtains a master's degree and is appointed as a teacher. However, she does not feel stable: “I had to support myself and didn't dare talk to anyone about my problems. It was survival.” After moving in with her boyfriend, she can no longer maintain that everything is going well. She notices a loss of control. “I often reported myself sick at work; I functioned less and less well.”

##### 3.2.1.2 Life Story After Treatment

The client realizes that she lost control much earlier in her life, around the age of 16. “Sudden outbursts of sadness, anger and fear often left me and those around me totally distraught. I had completely lost touch, with myself and with others.” She sees in retrospect that she had to use excessive control to keep herself going. “I needed more and more repression to keep control over my feelings.” She realizes that in the future, she will have to make a choice again and again, to avoid falling back into old behaviors. However, she is determined to achieve positive change. “But I know what I want in my life, and I want to work hard to achieve it.”

##### 3.2.1.3 Client's Perspective During Treatment

Client, in her initial evaluation (Week 6), is aware of the many ways in which she overcontrols and avoids. This strategy is so powerful that she does not experience her own emotions, and she herself is no longer aware of the person she is behind the strong and invulnerable woman she shows to others. She is more aware of her tendency to flee and avoid her emotions and her past. She is cautiously experimenting with allowing emotions in.

In recent weeks I have noticed (negative) emotions in myself for the first time. In addition, in recent weeks I have been expressing myself more often about my emotions, and what is on my mind. As a result, I increasingly notice that my role does not correspond with how I feel or even does not correspond with the facts that are available. This brings enormous uncertainty and panic‐like feelings.

The level of anxiety is evident from the fact that the client considered terminating the therapy in the first few weeks.

In her second evaluation (Week 18), client describes a complete lack of guidance and overview: “A description of the period between my first and second evaluation could be a tangle of yarn; everything is mixed up ….” Client describes that although her old strategies such as avoidance still regularly win out, she is now increasingly able to make a choice to experience and share her feelings with people around her.

On the third evaluation (Week 33), client sees that her agency has actually grown. She begins with the sentence, “[Name of institution] means choice. And making choices is exactly what I have done during my therapy process at [name institution].” She then describes numerous situations in which she has been able to make choices. She still has to choose again every day between seeking safety in avoidance or maintaining excessive control, and actually sharing and allowing the other person.

##### 3.2.1.4 Treatment Team Perspective During Treatment

At intake, strong self‐criticism, excessive control, and being stuck in work and social relationships are described. Both the anamnesis and psychiatric examination reveal a habit of pretending to be stronger. “[…] there is contact growth, though client may pretend to be seemingly competent.” Client's requests for help all relate to increasing her agency/self‐directedness. She would like to gain insight, be able to allow and regulate her emotions, and be able to be herself.

In the treatment team's first conclusion (Week 6), the team offers validation for the survival strategies the client has had to employ. “For a long time, the client put off thinking about the influence on her of the events that took place in her family of origin. She also had a good reason for this because she wanted to build her own life, get an education and build a career.” Next, the team invites her to practice new ways of dealing with feelings and with her environment. “You are very welcome to experiment and experience that there is more safety and security in your life today, more than you tend to think and you are used to experiencing.”

In the treatment team's second conclusion (Week 18), cautiously more self‐direction was noted. She practices letting go of her obsessive behavior and allowing and showing her emotions. Her struggle is seen, but she is encouraged to persevere and make choices that suit her. For example, even though it takes a lot out of her, client chooses to resume her work alongside therapy. “However, she indicates that she also wants to work towards a different balance at work and that she finds the support of the treatment program very important.”

The discharge letter concludes that client's excessive control has slowly given way to healthier self‐direction. “She practiced showing herself more, not only in her strengths but also in vulnerability, and paying attention to her feelings, no longer pushing them away or controlling them with food and avoidance.” The team sees that the client has gained insight into her pitfalls and knows what her challenges are. She is advised to continue her development with outpatient group therapy.

#### 3.2.2 Communion

##### 3.2.2.1 Life Story Before Treatment

In the first life story, client relates that both her parents struggled with severe mental health problems in her very early childhood (0–4 years). “Both were hospitalized or temporarily away from home during that period.” She and her brother were placed in varying places. Growing up, she exhibited strong peer contact, trying to keep her vulnerability and loneliness out of the picture. “I was told by fellow students afterwards that I always looked confident and unapproachable.” She describes her need to be seen and her attempts to capture attention with destructive behavior. “I was doing all kinds of impulsive acts, drinking too much, blowing, and self‐mutilating. All these things I stopped immediately when I got attention, either from parents of friends or from friends themselves.”

Later on, family therapy helped to improve contact with her parents, without becoming clear what concrete changes would occur. A turning point is moving in and living together with her boyfriend, which brings her vulnerability into focus. “He made me realize how seriously my life is determined by my fears and behaviors.” She mentions experiencing support and resolves to offer openness to those around her.

##### 3.2.2.2 Life Story After Treatment

Client realizes more the significance of missing her parents who were preoccupied with their own issues. “This gave me the idea that I was ‘broken’ or ‘defective’ and that I had to hide this as best I could, because otherwise my parents would leave me again.” Feeling insecure and anxious, she says she looked for ways to run away from these feelings. “My solution to this was to alienate everyone around me. Without connecting with others, I would also stop feeling lonely and abandoned.” As a turning point, she now mentions that her boyfriend had presented her with a choice: working towards change in therapy or ending the relationship.

She describes what treatment has brought her, “But I did learn to look with more mildness at the part of me that feels so lonely, abandoned, sad and angry. I can now acknowledge that it is allowed to exist.” And about connecting with others, “In addition, I have learned anew to connect with other people, and feel mutual affection.” She now has space for her own future in which she wants to continue working on her process and longs to connect with those around her and with children of her own. “I want to create a family situation that me and my parents didn't have,” she said.

##### 3.2.2.3 Client's Perspective During Treatment

In her first treatment evaluation (Week 6), client says she does not experience connection with herself. “[…] I got so absorbed in my role a long time ago that I forgot who I am myself.” In therapy, connection tentatively emerges when she first notices (negative) emotions in herself. “I am startled by my own insecurity, which I never actually felt. Not that I am afraid of these feelings, but they are new, confusing and overwhelming.” The threshold is high to share due to feelings of shame, but she does. “When I express my feelings in therapy, I find that it helps me a lot and I learn many things.” Together with a therapist, she works on planning to do fun things, which she does with her boyfriend despite feelings of guilt about them. No other people are mentioned in her treatment evaluation, including her parents.

In her second treatment evaluation (Week 18), client addresses her groupmates and the treatment staff with “Dear all.” She says she experiences more connection with herself and shares what she encounters with others. “I dare to express what is going on in me more often, whether it is sadness, irritation, or anger.” And she dares to look back. “Through sharing past experiences, I have begun to experience feelings around this.” She wants to be known more by those close to her. “First of all, I would like to practice making myself more show myself as I am as a whole, and not just the strengths.” There was hardly any room for relaxation earlier in her life but now she wants to explore what she enjoys.

In her third treatment evaluation (Week 33), the client describes important choices she made in her process. Her therapist asked her to either follow the full treatment or stop. “Then I made the first choice. I chose treatment, taking the first step on my path.” She then contemplates with understanding her desire to work anyway. “With this, I made a second choice, one that (in retrospect) did not work in my favor. But I couldn't do without it.” Next, she must continually relate to the other, and whether or not to connect. “The next choice I made consists of an infinite number of small choices, choices I still make every day. It is the choice between keeping people at a distance or letting people get close.” She concludes that she can look at herself and her early learning experiences with compassion. She expresses her gratitude to her practitioner and her groupmates for their part in her developmental process. “Before you, I had no one with whom I really connected, with whom I shared my worst and happiest moments.”

##### 3.2.2.4 Perspective of Treatment Team During Treatment

The intake report described severe psychological symptoms of both parents and tensions that eventually resulted in divorce. In the family, client did not learn to deal with or share her feelings. “Her parents had frequent fights, did not talk about feelings, were angry with each other and blamed each other a lot. After the arguments, they acted as if nothing had happened.” Her sensitivity and tendency to keep others at a distance by showing herself to be strong are described. “Client indicates she is hypersensitive with others and shuts down if she has done something she thinks the other person would not like. In addition, she is very critical of others.”

The treatment team's first conclusion (Week 6) acknowledges the instability and lack of safety and security in client's very early childhood. “Client does indicate that her parents later tried to repair the damage for their children. That is very important and helpful.” It is noted that client was already balancing on the edge of burnout for a long time. It is described that the client is still very much inclined to want to help others above all but that she is also gaining new experiences by showing herself more and sharing more, which is still very uncomfortable and frightening for her.

The treatment team's second conclusion (Week 18) notes that the client has become more in touch with her emotions. She has shared her feelings of emotional abandonment and explained in her group what makes connecting so exciting for her, both inside and outside of treatment. “A good thing is that client has this pattern of avoidance, a strategy to avoid disappointment and pain in an unstable environment, so clear. After all, that also creates room for making another choice.” Choices are left with her; she is allowed to focus her attention on what she wants and decide for herself whether to invite her parents and brother.

The discharge letter describes that the client has gradually managed to build up some trust in her fellow group members, the treatment team, and especially in her partner. It is seen that she has practiced showing herself more in her strength and vulnerability, and with standing still and tolerating her emotions, especially feelings of fear and uncertainty. Challenges include daring to allow more closeness, especially in the relationship with her partner, good self‐care (with regard to eating, working, and exercising), and staying in touch with her own emotions. “Client has thus gained important corrective experiences. She can continue to develop on the chosen path of trusting herself in a contact she experiences as reliable and stable.”

#### 3.2.3 Conclusion of Qualitative Analysis of the Themes of Agency and Communion

In her life story after treatment, the client reinterprets past events, gaining a new perspective on her relationships.

She realizes the severe loss of her parents and her overwhelming childhood loneliness. To cope, she had used excessive control to suppress her emotions. Now, she understands self‐direction differently; she no longer needs complete control and dares to connect with others. She recognizes that she has always played a role in her life, gradually forgetting her true self. This strategy protected her from hurt and uncertainty.

In therapy, she gradually connects with her feelings, noticing a discrepancy with her role. Initially, she runs from fear and uncertainty, but she learns that expressing feelings in therapy is beneficial. She describes her struggles with her fear of losing her control entirely, practicing new behaviors and often reverting to old strategies. Reflecting on her treatment, she identifies important choices that helped her take a new path, fostering self‐compassion and connection with others.

The treatment team sees increasing growth in healthy self‐direction over the course of treatment: a development from excessive control to learning to experience, tolerate, share, and process emotions, which increasingly offers client connection to herself and others.

## 4 Discussion

This study describes a psychotherapeutic process from multiple data sources in which, in addition to ROM data, the client's perspective and the treatment team's perspective through narrative data of the client and treatment team before, during and after treatment, were studied. The aim was to develop more understanding of psychotherapeutic change processes.

Due to a decrease in severity of personality problems and an increase in general functioning and well‐being after treatment and 1‐year following treatment termination, we can conclude that the single case study showed a successful treatment outcome. Important conditions (e.g., Livesley, Dimaggio, and Clarkin 2016; Dimaggio et al. 2018) seem to have been present for the efficacy of psychotherapy in the treatment offered: a recognized setting with a clear framework and method, safety and commitment, opportunity to connect with group and treatment team, promotion of mentalizing skills, encouragement of a new perspective on the self, attention to meaning, self‐compassion, making one's own choices, being allowed to struggle, attention to restoring contact ruptures, being able to practice behavior change, and experiencing connection, individuation and separation. The client's commitment significantly contributed to the therapy's success (Feinstein, Heiman, and Yager 2015).

The narrative psychological approach seems to offer good possibilities in better understanding change processes (Adler et al. 2012; Dimaggio et al. 2018; Gunderson 2018; Kealy and Ogrodniczuk 2018), linking the dimensions self (identity and self‐direction) and interpersonal (empathy and intimacy) functioning (LPF) to the narrative themes, agency, and communion (Adler et al. 2012; Lind, Adler, and Clark 2020; McAdams et al. 1996).

As aimed at Williams and Levitt (2007), treatment resulted in a significant increase in both agency and communion. These findings align with previous studies that found that agency increased in patients with personality pathology after psychotherapy treatment (Adler 2012; Lind et al. 2019, 2023; Pol et al. 2023; Timberlake and Fesel 2023). For communion, less unequivocal results were found, varying from no increase (Lind et al. 2019, 2023) to an increase in communion fulfillment (Adler 2012; Timberlake and Fesel 2023) and an increase in perceived quality of relationships (Pol et al. 2023). Furthermore, narrative data from the both the perspective of the client and the treatment team revealed an awareness of the need to balance the positive qualities (Niemiec 2019) of agency and community.

The current research showed that (a) new meaning was attributed to experiences in clients life story (autonomy does not mean controlling everything: awareness of excess of agency and lack of connection), (b) self‐insight emerged in treatment evaluations (consciously letting go of excess of control and experiencing a need for connection), and (c) development and behavioral change was noted in treatment team conclusions (growth in self‐directedness, and speaking out more and more, and eventually truly connecting with others). Despite the persistence of challenges, significant development seemed to have taken place in clients narrative identity, which is seen as essential for personal recovery in PD (Leamy et al. 2011; Sharp and Wall 2021). Notable in the qualitative results was the positive change in self‐understanding, which seemed to precede growth in self‐direction, and in experiencing connection and compassion with oneself, which seemed to provide support in connecting with others. These findings support the theory of Dimaggio et al. (2008), which suggest that self‐development and self‐reflection come before forming relationships with others.

### 4.1 Study Limitations and Future Directions

Single‐case methodology limits by definition generalizability. Moreover, the client's problems were within the specific indication area of intensive psychotherapeutic day clinic treatment. However, the case was representative of severe personality problems but less so in terms of ROM questionnaire completion. The baseline measurement of the MHC‐SF was missing because this instrument was not yet part of the standard ROM package. The qualitative content analysis focused on predetermined themes limiting broader but also unrestrained and less generalizable interpretations. Some other issues were underresearched; for example, there was no measurement of experiencing connectedness with oneself, whereas that can precede experiencing connectedness with others nor was there any measurement of the quality of the therapeutic relationship and perceived cohesion in the therapy group, despite these being important conditions for effective treatment (Feinstein, Heiman, and Yager 2015). However, the data show a clear development in relationships, both within the group and with the treatment team and in the partner relationship, which has resulted in an increase in connectedness.

For future research, the Agency‐Connectedness Scale (ACS‐30), a shortened version of the Agency Scale (Bekker 1993), could be added to the ROM. The theoretical background of the instrument is formed by a combination of object relationship theory and attachment theory (Bekker and van Assen 2006). A research design combining ROM data and narrative data could investigate change processes in a larger population and include different client and therapy characteristics.

In response to the demand for a better understanding of the efficacy of psychotherapeutic treatments, this single case study provided the opportunity to study the psychotherapeutic treatment process on a deeper level, using multiple data sources. The ROM data gained content and meaning through the use of narrative data, authentic material from the treatment context made accessible through quotations, supporting the idea of examining the development of narrative identity not only before and after psychotherapeutic treatment but also during treatment (Timberlake and Fesel 2023).

### 4.2 Concluding Remarks and Clinical Recommendations

This study provides some guidance for clinical recommendations: (a) It is not only valuable to use ROM data for treatment evaluation but also to include narrative data that includes the perspectives of both the client and practitioner, (b) realizing that narrative identity is at the core of personality pathology makes it valuable to actively support clients in reconstructing their narrative identity (for an example of a recovery focused narrative intervention, see Pol et al. 2024), (c) self‐development and self‐reflection seem to come before engaging with others and can be supportive in finding a balance between the positive qualities of agency and community, and (d) the realization that the LPF corresponds well with agency and communication can help in evaluating and developing treatment programs.

## Conflicts of Interest

The authors declare that the research was conducted in the absence of any commercial or financial relationships that could be construed as a potential conflict of interest.

## Data Availability

The data that support the findings of this study are available from the corresponding author upon reasonable request.
